# Microfluidic Processing of Piezoelectric and Magnetic Responsive Electroactive Microspheres

**DOI:** 10.1021/acsapm.2c00380

**Published:** 2022-07-29

**Authors:** Luís Amaro Martins, Joaquín Ródenas-Rochina, Daniel Salazar, Vanessa F. Cardoso, José Luis Gómez Ribelles, Senentxu Lanceros-Mendez

**Affiliations:** † CBIT—Centre for Biomaterials and Tissue Engineering, Universitat Politècnica de València, Valencia 46022, Spain; ‡ BCMaterials, Basque Center for Materials Applications and Nanostructures, UPV/EHU Science Park, Leioa 48940, Spain; § Department of Physics, Universidade do Minho, Braga 4710-057, Portugal; ∥ CMEMS-UMinho, Universidade do Minho, Guimarães 4800-058, Portugal; ⊥ Biomedical Research Networking Center on Bioengineering, Biomaterials, and Nanomedicine (CIBER-BBN), Madrid 28029, Spain; # IKERBASQUE, Basque Foundation for Science, Bilbao 48009, Spain

**Keywords:** microfluidics, microspheres, PVDF, cobalt ferrites, magnetoelectric

## Abstract

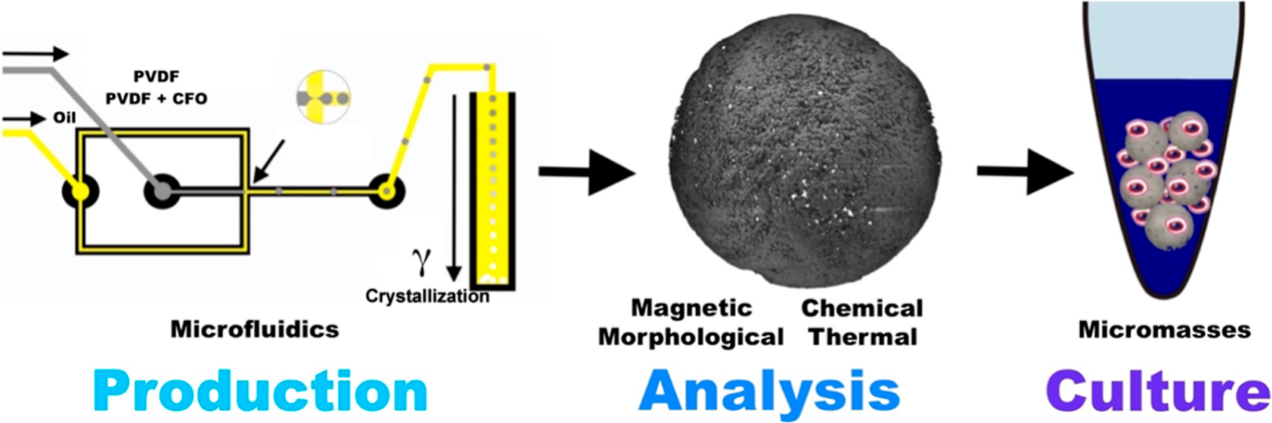

Poly(vinylidene fluoride) (PVDF) combined with cobalt ferrite (CFO) particles is one of the most common and effective polymeric magnetoelectric composites. Processing PVDF into its electroactive phase is a mandatory condition for featuring electroactive behavior and specific (post)processing may be needed to achieve this state, although electroactive phase crystallization is favored at processing temperatures below 60 °C. Different techniques are used to process PVDF–CFO nanocomposite structures into microspheres with high CFO dispersion, with microfluidics adding the advantages of high reproducibility, size tunability, and time and resource efficiency. In this work, magnetoelectric microspheres are produced in a one-step approach. We describe the production of high content electroactive phase PVDF and PVDF–CFO microspheres using microfluidic technology. A flow-focusing polydimethylsiloxane device is fabricated based on a 3D printed polylactic acid master, which enables the production of spherical microspheres with mean diameters ranging from 80 to 330 μm. The microspheres feature internal and external cavernous structures and good CFO distribution with an encapsulation efficacy of 80% and prove to be in the electroactive γ-phase with a mean content of 75%. The microspheres produced using this approach show suitable characteristics as active materials for tissue regeneration strategies and other piezoelectric polymer applications.

## Introduction

Polymers are strongly contributing to the development of smart and multifunctional materials. These can execute functions, respond to the environment, and exhibit sensory capabilities. Their application is wide, ranging from nanotechnology, biomimetics, neural networking, materials science, and molecular electronics, and future developments aim to develop technologies capable of mimicking the characteristics of muscle, brain, and nervous tissue.^1^

Piezoelectric polymers are of interest due to their ability to produce electric potentials through mechanical solicitation and vice versa.^2,3^ Among them, poly(vinylidene fluoride) (PVDF) gets particular attention due to its high piezoelectric constant, meaning maximal electromechanical transduction.^4^ Its intrinsic properties make it useful either as a sensor or as an actuator with no need for bulk and complex external wiring and power sources.^3,5^ PVDF is a semi-crystalline polymer with crystalline domains responsible for its piezoelectric behavior.^5^ When in the electroactive β- or γ-phases, its molecular arrangement promotes net permanent dipolar moments of the polymer chains, thus exhibiting its piezoelectric properties.^2^

On the other hand, when subjected to a magnetic field, magnetostrictive particles reorient their magnetic domains, changing their shape, allowing mechanical stimuli to be produced through magnetic solicitation.^6,7^ When embedded in a piezoelectric matrix, these particles enable the production of magnetoelectric composites by coupling their properties and allowing them to act as magnetoelectric transducers.^6,7^ Within the various magnetostrictive particles, cobalt ferrites (CFOs) exhibit high magnetostriction, chemical stability, and simple processability.^7^ The pair PVDF and CFO is therefore a great candidate for the production of polymer magnetoelectric composites.^8,9^

Electrical and mechanical stimulation are common practices in the biological and biomedical fields due to their influence on biological processes.^3,10^ For this reason, both PVDF and PVDF–CFO nanocomposites are largely explored as smart substrates for tissue engineering applications when electrical, mechanical, or even electromechanical stimulation can be easily provided in an airtight compartment.^2,3,11^

The ability of magnetoelectric compounds such as PVDF–CFO nanocomposites to transduce signals, namely, magnetic to electric, can be used to produce scaffolds for tissue engineering capable of dynamic electrical stimulation by generating surface electric potential variations upon magnetic solicitation.^12,13^ These functional 3D culture supports emulate the native environment and ECM properties of tissues which are highly dependent on electrical stimulus such as the nervous system, cardiac muscle, or bone.

Additionally, this can be achieved remotely through magnetic fields.^12,13^ The action of an applied magnetic field induces movement and deformation of CFO particles which, in turn, deforms the PVDF polymer matrix and originates an electric field in any point of a 3D culture by surface electric charge density variation at the surface of the microspheres, due to the piezoelectric properties of the material.^12,14^ It is worth noting that the piezoelectric properties of PVDF are originated by the polar arrangement of its crystal structure in β or γ crystalline forms. It is thus relevant that the crystallization of PVDF in the microspheres takes place preferentially in any of these phases.^15^

Both PVDF and PVDF–CFO have been widely explored as tissue engineering substrates in the form of membranes,^16−20^ fibers^21−26^ and, to a lesser extent, and not as widely explored, microspheres,^27−29^ or 3D substrates.^30−32^ Nevertheless, smart and functional microparticles with piezoelectric and magnetic properties are useful because cells and microparticles can be encapsulated, for instance, in a hydrogel, so that cells can be stimulated by mechanical stress and electric fields produced locally.

Both PVDF and PVDF–CFO nanocomposites have been processed by electrospray to form spheres with diameters in the order of a few microns,^7,33^ but present some limitations^7,34^ First, the control over microsphere size is difficult and broad size distribution often obtained. On the other hand, the microspheres are collected on a metallic surface on which they tend to agglomerate. The difficulty of collecting and dispersing the microspheres upon production sometimes hinders their applicability. PVDF and their composites have seldom been produced by other common techniques used to produce polymeric microspheres with only two references found, one for nanoprecipitation and one for emulsion.^35,36^

Microfluidics are miniature fluidic systems composed of reduced channel sizes that originate laminar flows, making them very well suited for the obtention of uniform polymer microspheres.^37,38^ These systems possess several advantages when compared to other common methods for polymer microsphere production.^37,38^ They can be automated, are fast, have high throughput, and allow narrow size microsphere distribution.^39,40^ Thus, microfluidic systems have been fabricated and applied to produce various types of polymer microspheres from polylactic acid, sodium alginate, and polycaprolactone, among others, but always unsuccessful when applied to PVDF.^41−43^

In this work, PVDF and PVDF–CFO microspheres are produced employing optimized microfluidic systems built in a simple and reproducible manner using low-cost materials and easily accessible microfluidic processing techniques.

## Materials and Methods

### Materials

PVDF (Solef 6010) and *N*,*N*-dimethylformamide (DMF), pure grade, were supplied by Solvay and Fluka, respectively. CFO nanoparticles (≈30–55 nm), Triton X-100, and PDMS (Sylgard 184) were purchased from Nanoamor, Sigma-Aldrich, and Dow Corning, respectively. Soy lecithin was acquired from Guinama. All chemicals were used as received from the suppliers.

### Microfluidics System Fabrication

**Figure 1:**
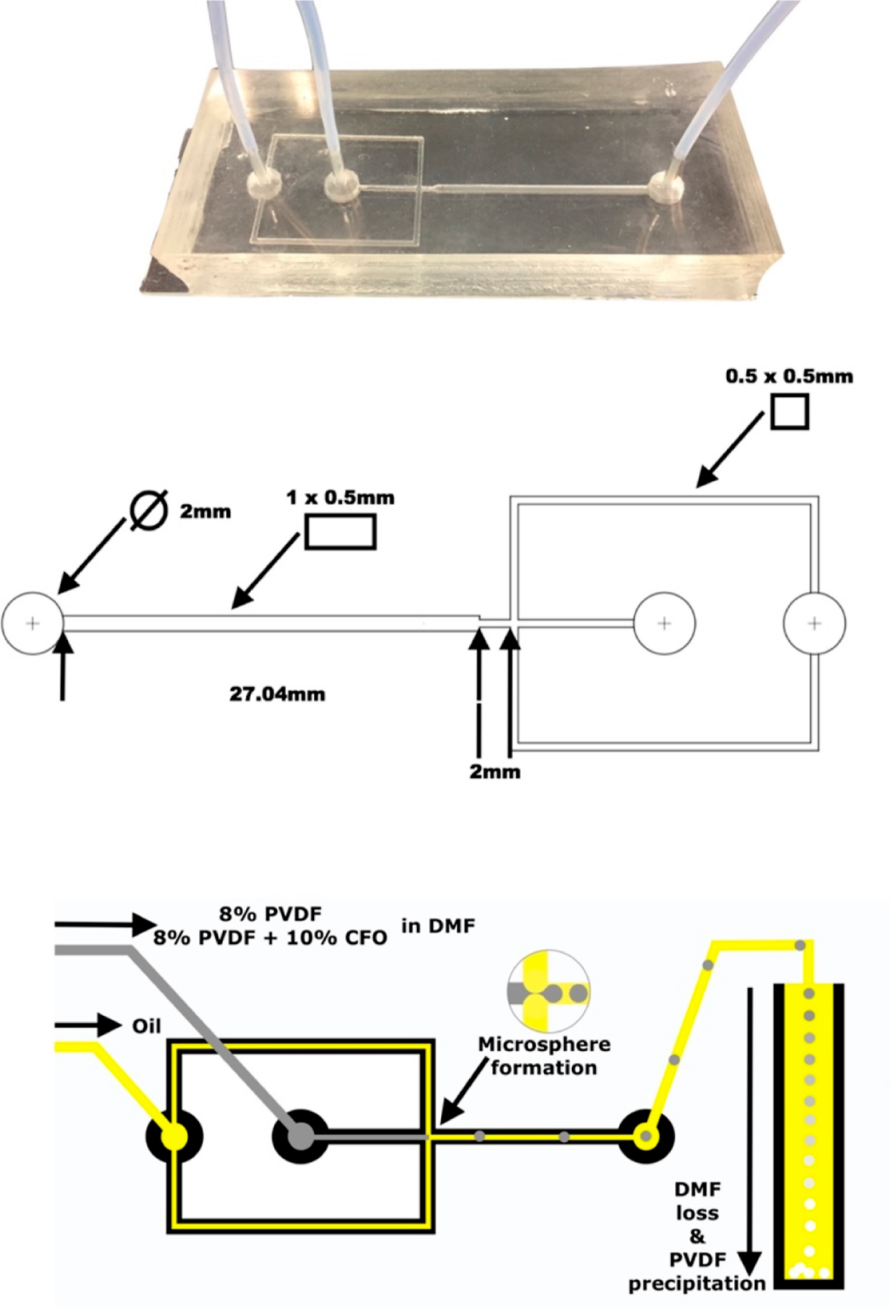
Microfluidic flow-focusing device, device’s channel dimension, and PVDF and PVDF–CFO microsphere production scheme (top to bottom).

Production of PDMS microfluidic systems was performed by a replica molding technique. First, microfluidic masters were drawn using free digital modeling software (FreeCAD), Figure 1.

The design was then processed in a slicing software (Cura) and 3D printed (Prusa i3 mk3) with polylactic acid (PLA) (FFFWorld PLATech) over a glass bed. PDMS and activator were thoroughly mixed in a standard 10:1 (w/w) ratio. Once degassed in a vacuum chamber, it was poured over the master’s and cured at 40 °C for 24 h. The structured PDMS slabs were then removed and cut to size, and the inlets and outlets punched with an appropriate tool. The PDMS slabs were irreversibly sealed to a glass slide by oxygen plasma (Piccolo, Plasma Electronics) following a standard procedure.^44^

PTFE tubes (1.6 and 0.5 mm external and internal diameters, respectively) were then inserted into the microfluidic system inlets and outlet and connected to plastic syringes. An Advanced Programmable Syringe Pump from Harvard Apparatus and a syringe pump NE-1010 from New Era Pump Systems were used to control the deployment of fluids into the PDMS microfluidic systems.

### Microparticle Processing

Sunflower seed oil with 1% (w/v) soy lecithin was used both as the continuous medium and as the gelifying bath.

For the discontinuous medium, two solutions were prepared to obtain either PVDF or PVDF–CFO microspheres. The concentrations of the polymer and filler were previously optimized to guarantee optimum processing conditions: for pure PVDF microspheres, an 8% (w/v) PVDF solution in DMF was prepared under magnetic stirring at 80 °C for 1 h to achieve fast dissolution.^20^ On the other hand, due to the magnetic nature of the CFO particles, the PVDF–CFO solution was first ultrasonicated in DMF for 1/2 h after which the surfactant Triton X-100, at a concentration of 0.02 g/mL, was added and left under sonication for an additional 3 and 1/2 h.^45^ The bath temperature was turned to 80 °C and PVDF was added to the solution. A mechanical PTFE stirrer was then inserted, and the solution was left for 1 h extra under both mechanical stirring and ultrasonication, ensuring complete polymer dissolution and magnetic particle dispersion. The final product was an 8% PVDF solution with 10% (w/w) CFO relative to the PVDF–CFO blend in DMF. Both solutions were cooled down to room temperature before use.

Syringes were filled with the corresponding continuous and discontinuous mediums, inserted into syringe pumps, and primed. After that, these were connected to the corresponding PTFE tubes and pumping was initiated. Continuous and discontinuous mediums were set between 0.05–0.25 and 0.004–0.020 mL/min, respectively, to change the pressures of the fluid promoting the systematic breakaway of smaller or bigger drops, thus defining the final microspheres dimensions. Droplet formation was unstable for values outside this ratio.

After production inside the microfluidic device, the microdroplets were dropped in a vertical glass column filled with the above-mentioned oil solution as exemplified in Figure 1. The microspheres were recovered from the bottom of the column after 24 h in order to ensure complete solvent removal. Once recovered, the microspheres were thoroughly washed with acetone first, to remove any oil remnants, and then water to remove any acetone residues. The process is completed by leaving them to dry overnight.

### Sample Characterization

Surface images were acquired in a Zeiss Ultra 55 field emission scanning electron microscope (FE-SEM) at 1 kV power without coating.

Internal images were taken with a Zeiss focused ion beam (FIB) Auriga Compact. Cuts were performed with an argon ion beam at 2 μA and the pictures were taken at 5 kV. To observe the interior of the microspheres, the samples were previously carbon coated in a Leica EM MED 020 sputter to enhance contrast.

Microsphere diameters were digitally measured with ImageJ analysis software. Pictures for the dimension assay were taken with a tabletop stereo microscope Leica MZ APO. Fifty microspheres were measured, and the results are provided as mean diameter and standard deviation.

Confocal microscopy images were acquired on a OLS3100-USS, LEXT model (Olympus 1) with a 408 nm laser diode and maximum resolution of 120 nm in *X*/*Y* and 10 nm in *Z*. Observations were taken using a 436 grid (four horizontal and six vertical sections), recorded at 4.84 mm^2^ with a maximum depth of 10 mm.

Fourier-transform infrared spectroscopy (FTIR) spectra were recorded in a Bruker Alpha II FTIR Spectrometer in the attenuated total reflectance mode. Spectra were recorded from 128 scans at 4 cm^–1^ resolution, between 400 and 4000 cm^–1^. Differential scanning calorimetry (DSC) thermograms were recorded on a PerkinElmer 8000 DSC. Samples were sealed in aluminum pans. A baseline, obtained by measuring an empty aluminum pan, was subtracted from the original sample thermograms. The analysis was performed under nitrogen (N_2_) purge at 20 mL/min from 30 to 200 °C at a 20 °C/min heating rate, as advised by the equipment manufacturer. Thermogravimetric analysis (TGA) was performed on a Mettler Toledo TGA analyzer with alumina pans used to hold the samples. The analysis was performed under a N_2_ atmosphere at 50 mL/min from 30 to 800 °C at a 10 °C/min heating rate in order to optimize the scan resolution. Magnetic properties were assayed at room temperature in a MicroSense EZ7-VSM vibrating sample magnetometer (VSM) from −18 to 18 kOe.

### Microspheres Disinfection and Preconditioning

Before cell culturing, the microspheres were disinfected with 70% ethanol to eliminate possible microorganisms. The protocol applied was as follows: PVDF microspheres were incubated with 100% ethanol for 10 min, followed by a double incubation in 70% ethanol for 10 min, and finally incubated overnight with 70% ethanol. To remove the ethanol, microspheres were washed for 10 min in decreasing ethanol/water dilutions, 50/50, 30/70, 10/90, and finally 0/100. To improve cell adhesion, materials were incubated with Dulbecco’s modified Eagle medium (DMEM) high glucose (Fisher) supplemented with 1% (v/v) of penicillin/streptomycin (Fisher) and 10%(v/v) fetal bovine serum (Gibco). This final step was carried out overnight in an incubator at 37 °C. All the steps above were performed with a Grant-Bio PTR-35 microcentrifuge tube vertical rotator.

### Cell Expansion and Cytotoxicity Assay

Microspheres metabolic and cytotoxic effects were evaluated through direct contact with rabbit chondrocytes (RC) by the [3-(4,5-dimethylthiazol-2-yl)-5-(3-carboxymethoxyphenyl)-2-(4-sulfophenyl)-2*H*-tetrazolium] (MTS) colorimetric assay, assessing the materials’ biocompatibility and CFO retention. RCs were used because they are a precursor line to cartilaginous tissue and one of the targets for the electrically stimulated tissue culture.^3,46^ RCs were expanded until passage 5 with DMEM high glucose culture medium (Fisher) enriched with 1% (v/v) of penicillin/streptomycin (Fisher), 1% (v/v) sodium pyruvate (Fisher), 10% (v/v) of fetal bovine serum (Gibco), and 50 μg/mL ascorbic acid (Sigma-Aldrich), and cells were collected and concentrated to 0.4 × 10^6^ cells/mL and combined with 0.02% (v/v) PVDF (66 μm) or PVDF–CFO (52 μm) microspheres. Cells with PVDF, PVDF–CFO, or only cells (control) were seeded by transferring 500 μL of the cell suspension to 500 μL microtubes and centrifuged at 1000 rpm for 5 min to allow micromasses formation with a final diameter of around 1 mm. Cell micromasses were cultured at 37 °C and 5% CO_2_. At 24 and 72 h, three samples of each group were removed, washed with Dulbecco’s phosphate-buffered saline, and placed in a 24 well plate. Each sample and control were covered with 400 μL of MTS reagent diluted in DMEM high glucose (1:10) without phenol red and incubated at 37 °C and 5% CO_2_ for 2 h and 30 min. As the inner control, three empty culture wells were filled with 400 μL of MTS reagent. Finally, colorimetric measurements of the formazan dye were performed on a Victor3 spectrometer at 490 nm. Results were expressed as relative MTS activity as compared to control conditions. All assays were performed in triplicates to ensure their statistical relevance.

## Results and Discussion

Flow-focusing microfluidic systems use an immiscible fluid to systematically break the polymer solution into small droplets. These naturally take a spherical shape due to its isotropic nature, as fluids tending to reach equilibrium minimizing the system’s free energy and achieving thermodynamic stability.^47,48^ The channels dimensions in the micrometer range guarantee a continuous laminar flow, thus producing monodisperse droplets.

Polymer precipitation is achieved through phase inversion because DMF and sunflower oil mix slowly, while still allowing droplet formation at the channels crossing point. Droplets are then dropped on an oil-filled vertical column, the spherical shape is maintained, while the polymer slowly precipitates and crystallizes due to the gradual solvent diffusion to the oil medium, as exemplified in Figure 1, reaching the bottom as solid polymer microspheres.

### Morphological Analysis

**Figure 2:**
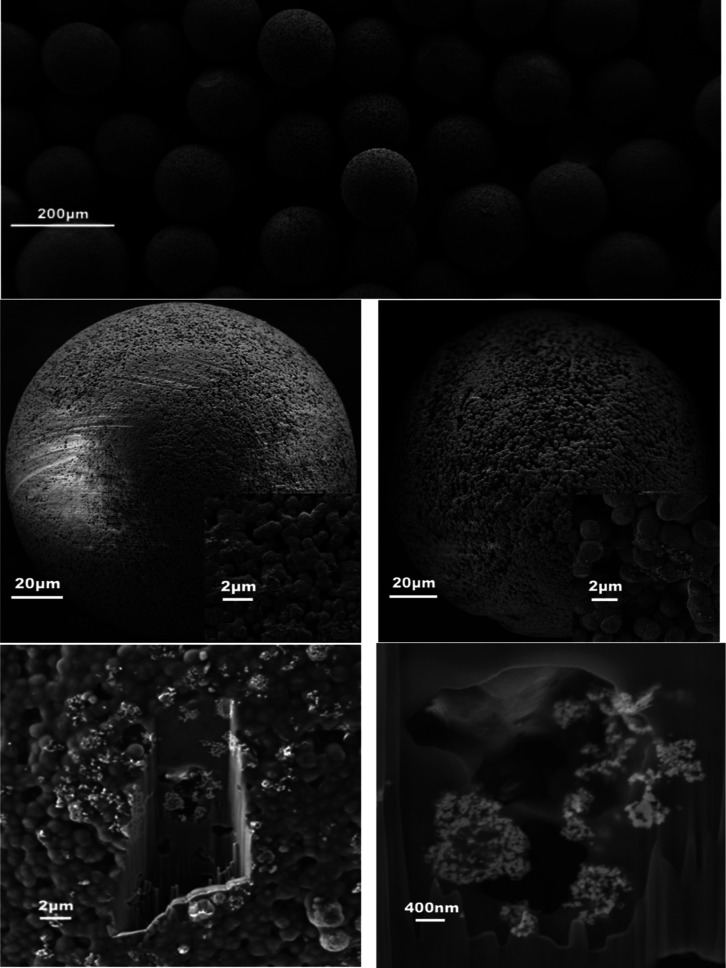
FESEM images of various PVDF microspheres (top), PVDF, and PVDF–CFO microspheres, with insets of surface details (center left and center right, respectively), and a FIB cross-sectioned PVDF–CFO microsphere (bottom left and bottom right).

Representative FESEM and FIB images of PVDF and PVDF–CFO microspheres processed by microfluidic technology are presented in Figure 2.

Both samples, PVDF and PVDF–CFO, exhibit a spherical shape without visible defects. The surfaces, detailed in the insets, feature a textured and porous morphology in both samples, resembling an aggregate of micron size microspheres. These structures are similar to PVDF spherulites formed from solution, as occurred in PVDF membranes obtained by non-solvent-induced phase separation using a low-temperature coagulation bath.^49,50^ On PVDF–CFO samples, CFO is presented on the surface of the microspheres, as can be seen on the inset of Figure 2. Distribution is even with small CFO nanoparticle clusters uniformly distributed throughout the surface.

The FIB cross-sectioned FESEM images of the PVDF–CFO microspheres, Figure 2, shows that CFO nanoparticle distribution is organized in small clusters spread throughout the sample. Thus, both surface and cross-sectional images indicate good particle dispersion. Moreover, pores can be observed on the inside and surface of the microspheres, indicating a cavernous internal structure extending all the way through the microsphere. Confocal microscopy images of PVDF–CFO microspheres, Figures S1 and S2, add a more detailed look on surface morphology and crystallite structures and are available as Supporting Information.

The microfluidic system produces microspheres in a wide range of sizes from 66 to 174 μm for PVDF and 52 to 235 μm for PVDF–CFO by tuning the flow ratio of continuous to discontinuous mediums between 62.5 and 2.5, respectively. Higher ratios between continuous and discontinuous media produce microspheres with smaller dimensions. Below and above said ratios, the system’s behavior became instable and unable to produce droplets or the droplets spherical shape was lost by colliding with the system walls.

**Figure 3:**
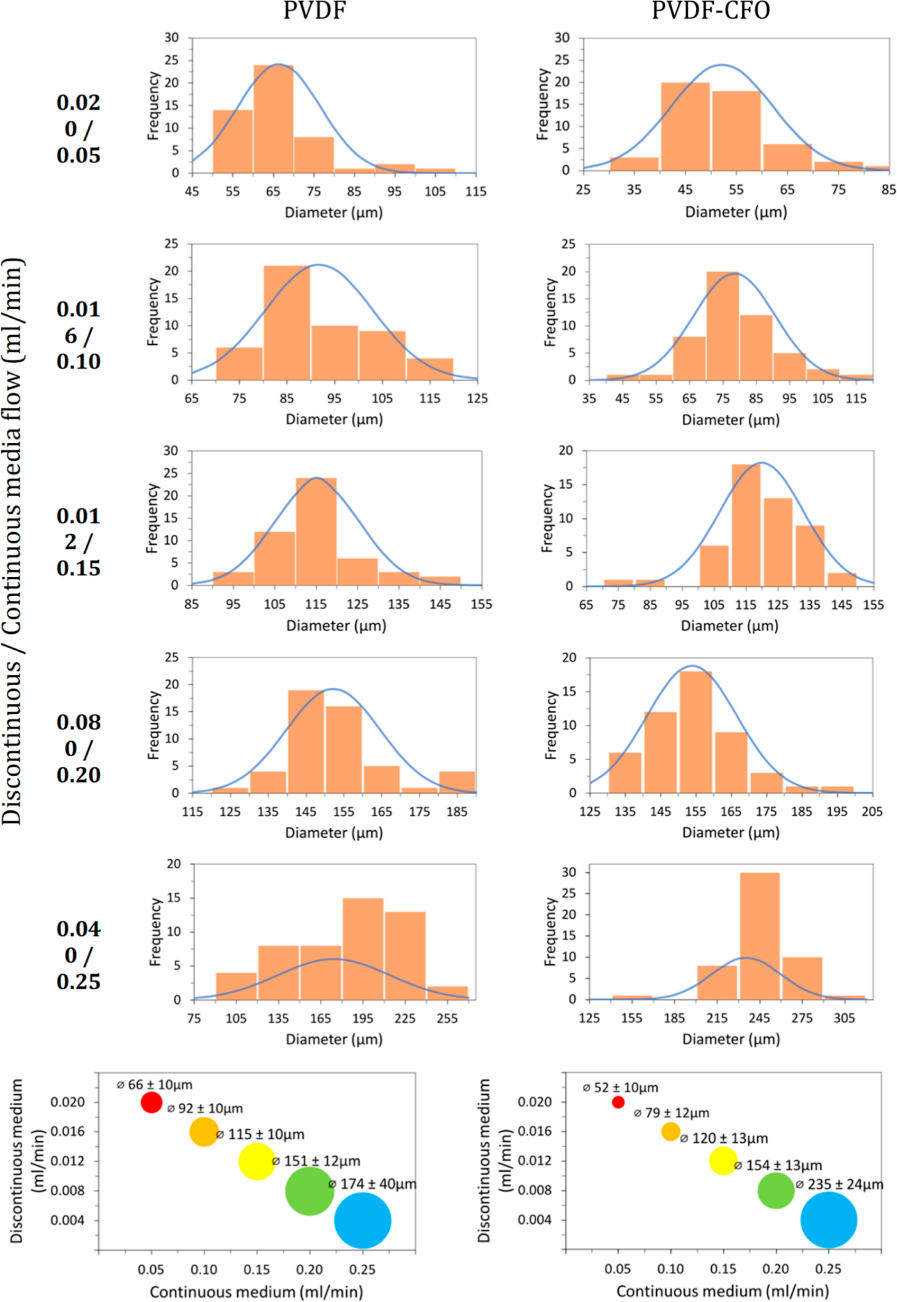
Histograms of PVDF and PVDF–CFO microsphere diameter for the diverse processing parameters (*n* = 50) (top), microsphere diameter dependence on processing parameters (bottom).

Size distribution, average size, and standard deviation of the microspheres produced are exhibited in Figure 3. Differences in microsphere diameter between PVDF and PVDF–CFO samples have been observed for the same processing conditions, probably due to the magnetic attraction among CFO particles altering solution properties and behavior. Before collection, microspheres were sieved in order to remove the largest aggregates.

The produced microspheres did not achieve the level of monodispersity commonly associated with microfluidics, although dispersity is far superior to the size distribution of electrosprayed microspheres.^7^ Because the system operates under a laminar flow regime, microdroplet separation occurs in a systematic and repeatable manner, generating monodisperse droplets, but coalescence happens due to the flowing nature of the continuous medium and the static nature of the precipitation column making droplets experiencing a flow reduction and coalescence while going from one to the other. Coalesced droplets dropped faster and consequently coalesced with other non-precipitated droplets on their way, leading to a snow-man effect. This fact could be avoided by automating the system or manually distributing the droplets through the column surface, leading to increased monodispersity.

### Physical and Chemical Characteristics

**Figure 4:**
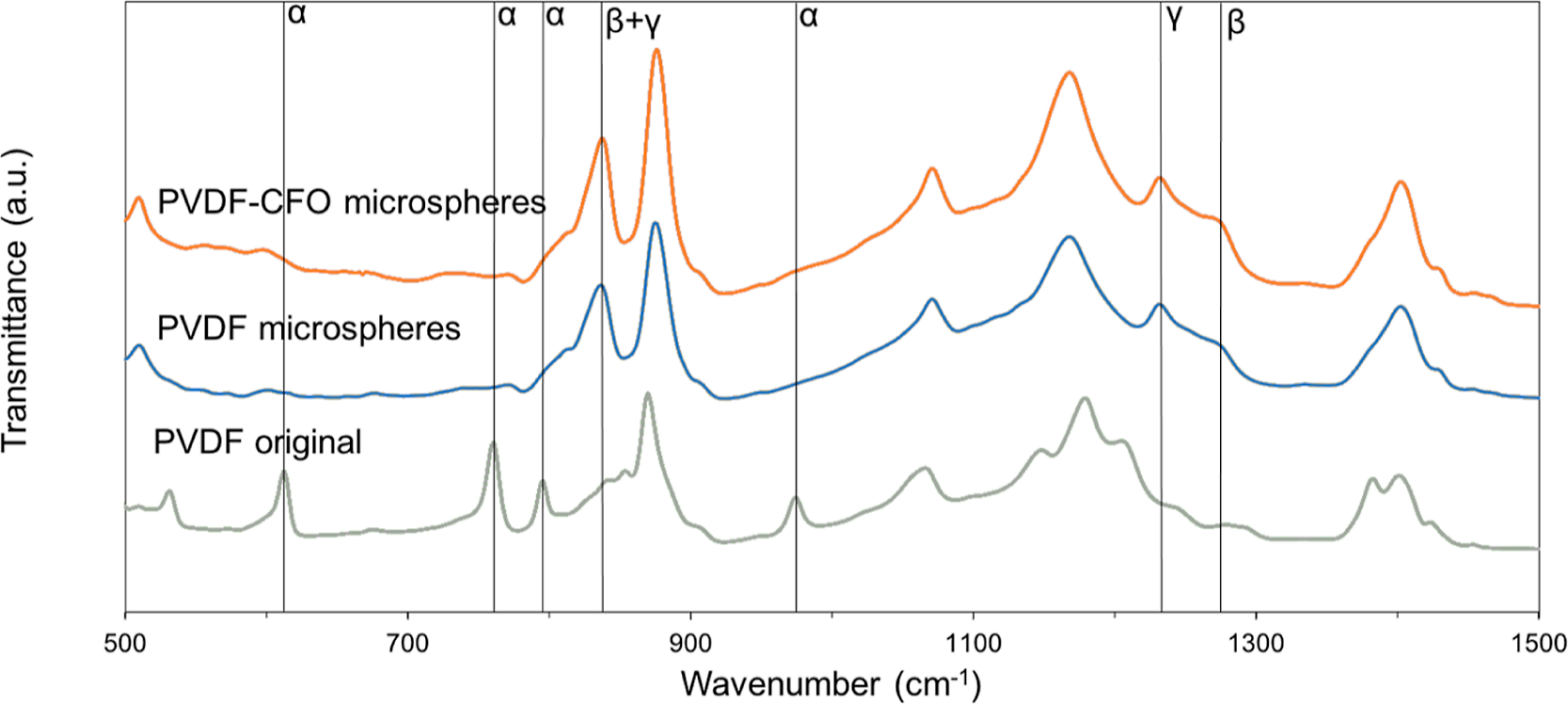
FTIR spectra for the PVDF and PVDF–CFO microspheres. Original PVDF pellet spectrum is also shown for comparison.

The infrared spectra (IR) of the PVDF and PVDF–CFO microspheres can be observed in Figure 4. The spectrum of the original PVDF pellets is also included. The polymers’ chemical structure is composed of repeating −CH_2_–CF_2_– units, and α, β, and γ-phases identification is possible by their characteristic vibrational modes described in Table 1.^33,51,52^

**Table 1:** PVDF and Corresponding Crystal Phase IR Specific Peaks

crystalline structure	characteristic peaks (cm^–1^)	chemical group
α-phase	614	CF_2_
	763	CF_2_
	795	CH_2_
	975	CH
β-phase	1276	CF
	840	CH_2_
γ-phase	1234	CF_2_

The characteristic peaks of the α-phase are clearly shown in the original PVDF, which shows intense peaks at 614 and 763 cm^–1^, Figure 4.^51^ Nevertheless, in PVDF microspheres with or without CFO, the predominant phase is the γ-phase, as observed by the presence of the intense 1234 cm^–1^ peak.^51,52^

The fractions of α, and electroactive (EA) phase, β, and γ, can be determined using eq 1
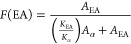
1where *F*(EA) is the percentage of the EA phase, *A*_α_ and *A*_EA_ are the baseline-corrected absorbances at 766 and 840 cm^–1^, respectively, and *K*_α_ and *K*_EA_ are the absorbance coefficients of the respective peaks at 6.1 × 10^4^ and 7.7 × 10^4^ cm/mol.^7,53^

**Figure 5:**
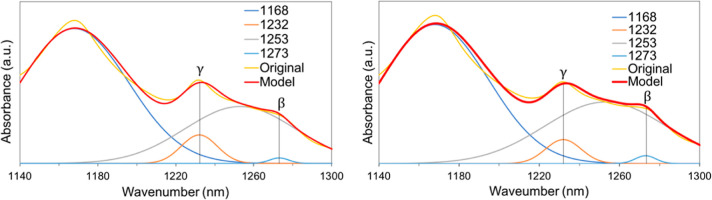
FTIR spectra deconvolution for PVDF (left) and PVDF–CFO microspheres between 1140 and 1300 cm^–1^ (right).

A deconvolution of the FTIR spectra in the regions between 1140 and 1300 cm^–1^ for the PVDF and PVDF–CFO microspheres was performed to isolate specific β and γ-phase peaks, as shown in Figure 5.

The percentages of individual contributions of β and γ structures to the EA phase can be calculated by eq 2
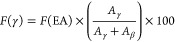
2where *F*(γ) is the γ-phase percentage, *F*(EA) is the EA-phase percentage, and *A*_γ_ and *A*_β_ are the 1234 and 1275 cm^–1^ peaks’ relative absorption intensities.^51^

PVDF and PVDF–CFO microspheres are mainly crystallized in the γ-phase. This phase represents a polar arrangement of the polymer structure where the highly electronegative F forms a C–F bond with significant dipole moment originating its piezoelectric effect. This phase is characterized by an orthorhombic unit cell with a TTTGTTTG′ sequence of trans and gauche conformation.^54,55^

Solvent type, hydrostatic pressure, and presence of surfactants are known to modulate crystallization kinetics and induce γ-phase crystallization in PVDF films.^56,57^ On PVDF films processed by the NIPS method (similar to the developed microfluidics precipitation method), γ, β, and α phases can be produced independently of the diffusion rate. Solvent-antisolvent dipole-moment interactions are deemed to be the major factor affecting PVDF crystallization. Increasing dipole-moment sequentially produced α, γ, and β structures, while it was common for samples to exhibit a combination of them.^58^

In order to yield such high values of γ-phase, PVDF processing techniques commonly rely on high temperatures, additives, or post-processing. Furthermore, these processes are only applied to films and would be particularly difficult to apply to other morphologies, especially microspheres, due to the lack of flat surfaces.^52,59^

In the analysis of the FTIR spectrum shown in Figure 5, we provide a comparison between the ratio of γ to β peaks intensity in the different samples, given by *F*(γ) in eq 2. Meaningful information can be withdrawn from the relative peaks positioning and intensity rather than absolute peak intensity.

It can be observed that the presence of CFO nanoparticles increases slightly *F*(γ) from 73 to 75%. This slight difference in the γ-phase content between samples is possibly explained by the interaction between the negative zeta potential of the CFO particles and the positively charged CH_2_ groups increasing γ-phase nucleation.^9,60,61^ The mechanism seems valid for crystallization in a polar arrangement, β or γ in the presence of positively charged particles contributing to the polymer’s polar arrangement.

**Figure 6:**
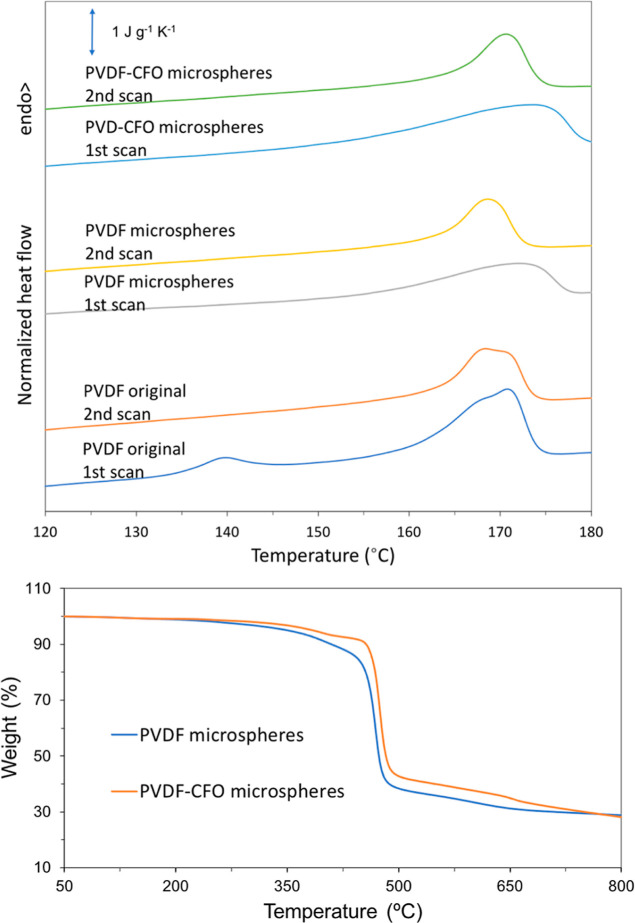
DSC spectra for the PVDF and PVDF–CFO microspheres (top) and TGA spectra for the PVDF and PVDF–CFO microspheres (bottom).

Thermal properties of the PVDF and PVDF–CFO samples were studied by DSC calorimetric curves and analyzed in terms of their thermal parameters: melting temperature (*T*_m_), melting enthalpy (Δ*H*_s_), and the crystalline fraction (*X*_c_). The melting process is characteristically observed in DSC calorimetry as an endothermic peak. *T*_m_ was established as the peak’s maximum, as in Figure 6, and *X*_c_ was calculated according to eq 3
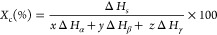
3where *X*_c_ stands for the sample crystalline fraction, Δ*H*_s_ for the PVDF melting enthalpy, corresponding to the melting peak’s area, x, y, and z stand for the percentage of α, β, and γ-phases, respectively, and were calculated by eqs 2 and 3, Δ*H*_α_, Δ*H*_β_, and Δ*H*_γ_ are the melting enthalpies for a 100% crystalline sample in the respective phases. Δ*H*_α_, Δ*H*_β_, and Δ*H*_γ_ were considered as 93.07, 103.40, and 104.60 J/g, respectively.^61,62^ Thermal parameters are summarized in Table 2.

**Table 2:** Thermal Parameters and Content of Crystal Phases of PVDF and PVDF–CFO Microspheres

	PVDF	PVDF–CFO
*T*_m_ (°C)	171	172
Δ*H*_s_ (J/g)	44	64
*F*_α_ (%)	12	10
*F*_β_ (%)	15	15
*F*_γ_ (%)	73	75
*X*_c_ (%)	44	63

All thermograms are characterized by a single endothermic peak. *T*_m_ is low for γ crystalline structures that usually melt at higher temperatures, probably due to crystallization at room temperature because much higher temperatures are usually used to achieve γ-phase crystallization.^52^

First and second heat scans behave similarly whether PVDF or PVDF–CFO are regarded. Three main differences can be observed when comparing the melting peaks from structures crystallized from solution and from the melt. Solution crystallized peaks are wider, lower in energy, and have higher *T*_m_. These reflect the differences in the crystalline structure under different crystallization conditions.

Wider peaks indicate a distribution of crystallites with different sizes in the solution crystallized samples. On the other hand, *T*_m_ is dependent on crystallites’ lamellae thickness, confirming different crystal sizes forming from the melt or solution, as previously mentioned. The higher fusion enthalpy of the first heat fusion peak correlates to the presence of more ordered structures and thus less amorphous portions and higher crystallinity.^63^

It is frequent that double peaks appear in the DSC heating scans of PVDF^64^ and other semicrystalline polymers.^65^ They can be ascribed not only to the presence of different co-existing crystalline phases but also to the recrystallization of the polymer chains during the heating scan itself. Crystallization at low temperatures produces small lamellae that in turn melt at low temperatures. During the heating scan, melting starts at temperatures still in the range of temperatures that allow crystallization. When this happens, an exothermal crystallization peak overlaps on the melting endotherm and the results seem to be a double melting peak.^66^

One can only speculate about the ground polymer powder because the thermal history of its crystallization process is not known. It is interesting to observe the melting endotherm of the microspheres in the first heating scan: although the microspheres are formed in the microfluidics process at room temperature (so with a very low crystallization temperature) the melting endotherm appears as a broad peak with a maximum temperature higher than in the original PVDF, and higher than in the second scan in which PVDF chains crystallizes, preferentially in α-phase. The previous discussion can create the expectation that melting starts in the heating scan at lower temperatures than in the second one. The observed behavior is an additional proof that in the microspheres PVDF is crystallized in the γ-phase which has a melting temperature higher than the α-phase.^52,67^

*T*_m_ is around the same value for both samples and in accordance with other articles.^7^ This way it is possible to conclude that neither processing in a microfluidics system nor the addition of 10% CFO pose any significant changes in the polymer’s *T*_m_.^7,62^

Differences in *X*_c_ can be correlated with CFO addition due to CFO presence acting as nucleation centers for crystallite formation in PVDF.^7,60,68^

In Figure 6, the typical PVDF two-step degradation curve can be observed for both samples as stated elsewhere.^7,69^ The PVDF sample exhibited a lower onset temperature, 463 °C, relative to PVDF–CFO, 475 °C, revealing increased thermal stability with CFO addition, as previously mentioned.^7,70^ The degradation step between ≈370 and ≈470 °C in PVDF–CFO is attributed to the degradation of Triton X-100 remaining in the microspheres, thus being absent in the PVDF sample.^71^ The remaining mass can be explained by the non-volatile residues left after polymer pyrolysis.

**Figure 7:**
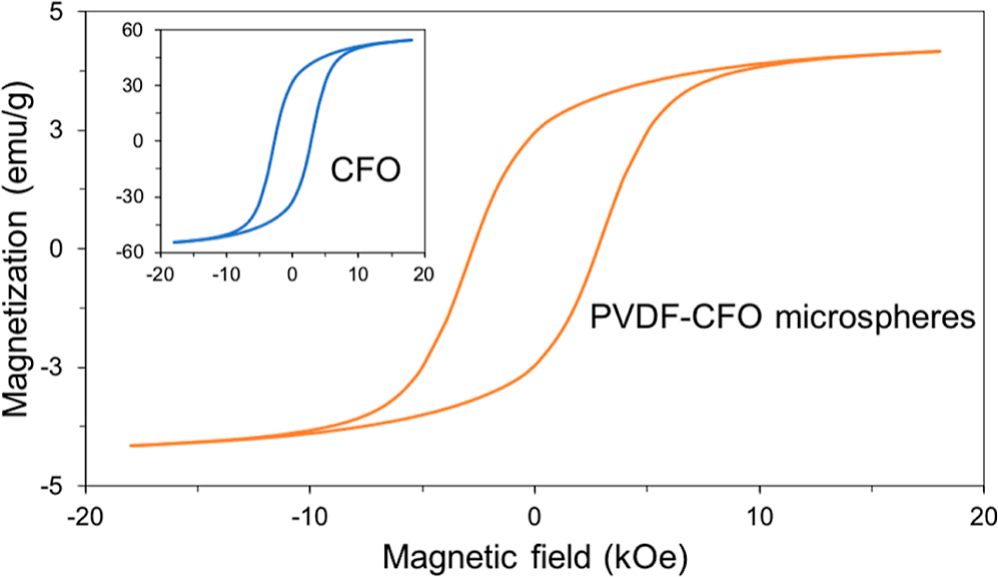
Hysteresis loop for PVDF–CFO microspheres and inset for pure CFO.

The hysteresis loop obtained at room temperature for the PVDF–CFO microspheres (Figure 7) revealed a magnetization of 4.3 emu/g, coercivity (Hc) of 2.8 kOe, and ferromagnetic behavior, as expected (Figure 7 on the inset). The hysteresis loop for pure CFO particles is also present, revealing a magnetization of 54.5 emu/g, and coercivity (Hc) of 2.6 kOe. Similar values of Hc indicate that the CFO nanoparticles were not modified or degraded during the processing of the PVDF–CFO microspheres. Mass percentage *M*_p_ of magnetic particles in the sample was calculated according to eq 4, where *M*_c_ is the normalized saturation magnetization of the pure CFO particles and *M*_s_ is the sample’s normalized saturation magnetization.
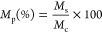
4

Considering the saturation magnetization values of pure CFO and PVDF–CFO samples, the mass percentage of magnetic particles in the sample was calculated to be 8%. Encapsulation efficacy (*E*_e_) expresses a measurement, in %, of CFO particle incorporation into the polymer matrix during microsphere processing. It can be calculated from eq 5, where *M*_p_ stands for the mass percentage of magnetic particles in the sample and *M*_pt_ stands for the mass percentage of magnetic particles incorporated into the polymer solution before processing. The *E*_e_ of PVDF–CFO processing is 80%, which is correlated to a 20% particle loss during processing, as magnetic particles tend to form dense aggregates that precipitate at the syringe bottom. Previous works of PVDF–CFO composite microspheres processed through electrospray exhibit particle loss in the order of 50%, showing the superior particle retention of the developed processing method.^7^
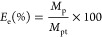
5

### Metabolic Activity Assay

**Figure 8:**
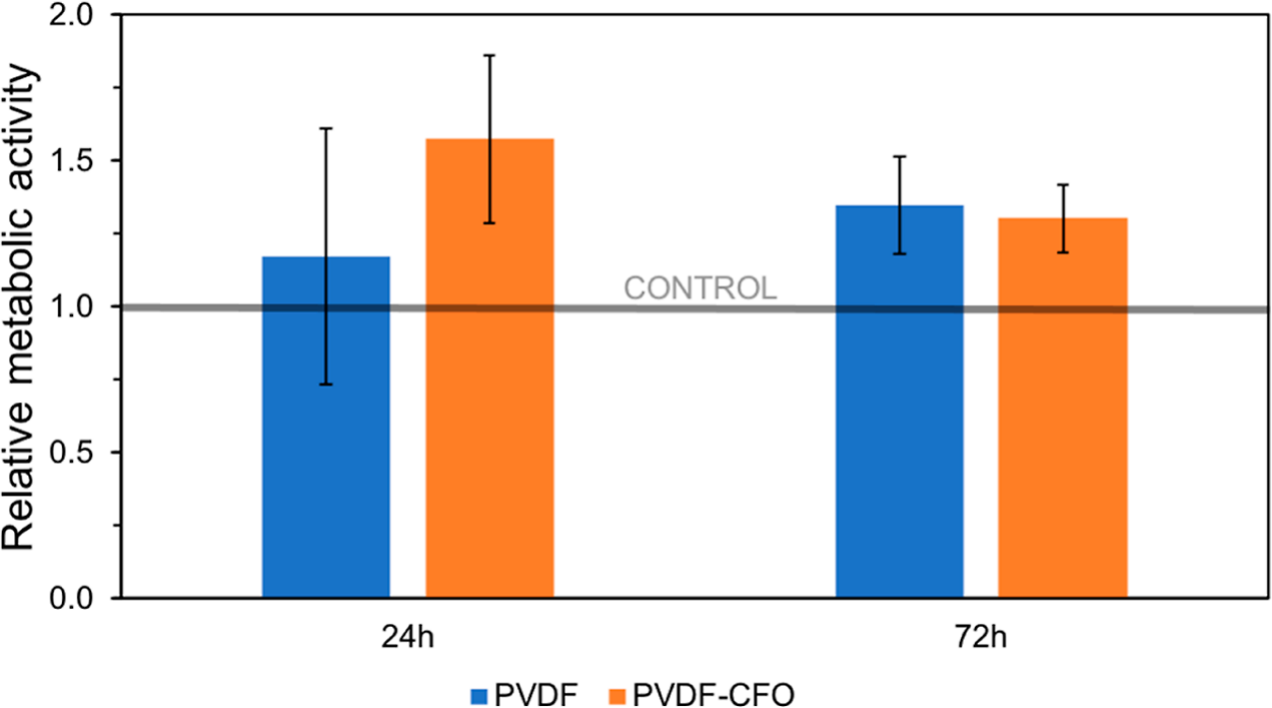
MTS relative metabolic activity for PVDF and PVDF–CFO microspheres at 24 and 72 h culture times.

MTS results of 3D cultured RC micromasses are summarized in Figure 8. RC micromasses surpassed the control’s metabolic activity when cultured with PVDF and PVDF–CFO microspheres for both 24 and 72 h assays. MTS values for the 24 h assay registered values of 1.2- and 1.6-fold the control for PVDF and PVDF–CFO, respectively. The 72 h assays registered closer values of approximately 1.3-fold for both PVDF and PVDF–CFO. The later assay also registered lower standard deviation values than the former.

Metabolic activity of RC was improved by a minimum of 1.2-fold in the presence of PVDF or PVDF–CFO microspheres, thus demonstrating its enhancement relative to the control (microsphere absence), which proves the materials’ cytocompatibility on direct contact. Although CFO’s cytotoxic effects are described in the literature, in our case, no negative effects were observed even though CFO clusters were observed on the PVDF–CFO microsphere’s outer and inner surfaces (Figure 2), thus proving the strong encapsulation/retention provided by the polymer’s matrix during culture conditions.^72,73^

## Conclusions

The proposed method based on microfluidic technology enables the production of narrow size dispersion PVDF and PVDF–CFO nanocomposite microspheres with a size range between 52 and 235 μm. The obtained microspheres exhibited a homogeneous and round shape and textured surface with good CFO particle dispersion and retention efficacy of 80%. Moreover, the samples exhibit high electroactive γ-phase content of 75%, and crystallinity between 44 and 64%, thus not requiring post-processing thermal treatments. Other thermal and chemical properties are on par with conventional processing methods. 3D culture of RC micromasses promoted an 1.2–1.7 fold increase in metabolic activity in the presence of PVDF and PVDF–CFO microspheres.

The proposed microfluidic approach is therefore a simple and cost- and time-effective solution to produce highly electroactive PVDF and PVDF–CFO microspheres in large quantities in an autonomous manner that is easy to collect and manipulate. The systems are fabricated using an easily accessible 3D printer and replica molding techniques available in most laboratories.

Our method provides a process for an easy production of microspheres with characteristics that aid development and research on the domain of piezoelectric polymers and the underlying areas of application.

## Data Availability

The raw data required to reproduce these findings cannot be shared at this time due to technical or time limitations.

## Supporting Information Available

Confocal microscopy images representing the surface of PVDF–CFO microspheres (PDF)

## References

[ref1] Gandhi M. V.; Thompson B. D. Smart Materials and Structures; Springer Science & Business Media, 1992.

[ref2] Ramadan K. S.; Sameoto D.; Evoy S. A Review of Piezoelectric Polymers as Functional Materials for Electromechanical Transducers. Smart Mater. Struct. 2014, 23, 033001 10.1088/0964-1726/23/3/033001.

[ref3] Ribeiro C.; Sencadas V.; Correia D. M.; Lanceros-Méndez S. Piezoelectric Polymers as Biomaterials for Tissue Engineering Applications. Colloids Surf., B 2015, 136, 46–55. 10.1016/j.colsurfb.2015.08.043. 26355812

[ref4] Fukada E. Recent developments of polar piezoelectric polymers. Electr. Insul. 2006, 13, 1110–1119. 10.1109/tdei.2006.247839.

[ref5] Sencadas V.; Gregorio R.; Lanceros-Méndez S. α to β Phase Transformation and Microestructural Changes of PVDF Films Induced by Uniaxial Stretch. J. Macromol. Sci., Part B: Phys. 2009, 48, 514–525. 10.1080/00222340902837527.

[ref6] Srinivasan G. Magnetoelectric Composites. Annu. Rev. Mater. Res. 2010, 40, 153–178. 10.1146/annurev-matsci-070909-104459.

[ref7] Gonçalves R.; Martins P.; Correia D. M.; Sencadas V.; Vilas J. L.; Leon L. M.; Botelho V.; Lanceros-Mendez S. Development of Magnetoelectric CoFe2O4/Poly (vinylidene fluoride) Microspheres. RSC Adv. 2015, 45, 35852–35857. 10.1039/c5ra04409j.

[ref8] Amiri S.; Shokrollahi H. The Role of Cobalt Ferrite Magnetic Nanoparticles in Medical Science. Mater. Sci. Eng., C 2013, 33, 1–8. 10.1016/j.msec.2012.09.003. 25428034

[ref9] Gonçalves R.; Martins P.; Moya X.; Ghidini M.; Sencadas V.; Botelho G.; Mathur N. D.; Lanceros-Mendez S. Magnetoelectric CoFe2O4/Polyvinylidene Fluoride Electrospun Nanofibres. Nanoscale 2015, 7, 8058 10.1039/c5nr00453e. 25871851

[ref10] Rajabi A. H.; Jaffe M.; Arinzeh T. L. Piezoelectric Materials for Tissue Regeneration: A Review. Acta Biomater. 2015, 24, 12–23. 10.1016/j.actbio.2015.07.010. 26162587

[ref11] Ribeiro C.; Correia D. M.; Ribeiro S.; Sencadas V.; Botelho G.; Lanceros-Méndez S. Piezoelectric poly(vinylidene fluoride) microstructure and poling state in active tissue engineering. Eng. Life Sci. 2015, 15, 351–356. 10.1002/elsc.201400144.

[ref12] Ribeiro C.; Correia V.; Martins P.; Gama F. M.; Lanceros-Mendez S. Proving the Suitability of Magnetoelectric Stimuli for Tissue Engineering Applications. Colloids Surf., B 2016, 140, 430–436. 10.1016/j.colsurfb.2015.12.055. 26797659

[ref13] Ferson N. D.; Uhl A. M.; Andrew J. S. Piezoelectric and Magnetoelectric Scaffolds for Tissue Regeneration and Biomedicine: A Review. IEEE Trans. Son. Ultrason. 2021, 68, 229–241. 10.1109/tuffc.2020.3020283. 32866097

[ref14] Reis S.; Silva M.; Martins P.; Lanceros-Mendez S. Applications of Polymer-Based Magnetoelectric Materials: Sensors. Actuators, Antennas, and Memories; Wiley-VCH Verlag GmbH & Co., 2017.

[ref15] Martins P.; Lanceros-Méndez S. Polymer-Based Magnetoelectric Materials. Adv. Funct. Mater. 2013, 23, 3371–3385. 10.1002/adfm.201202780.

[ref16] Ribeiro C.; Panadero J. A.; Sencadas V.; Lanceros-Méndez S.; Tamaño M. N.; Moratal D.; Salmerón-Sánchez M.; Gómez Ribelles J. L. Fibronectin adsorption and cell response on electroactive poly(vinylidene fluoride) films. Biomed. Mater. 2012, 7, 035004 10.1088/1748-6041/7/3/035004. 22356773

[ref17] Martins P. M.; Ribeiro S.; Ribeiro C.; Sencadas V.; Gomes A. C.; Gama F. M.; Lanceros-Méndez S. Effect of Poling State and Morphology of Piezoelectric Poly (Vinylidene fluoride) Membranes for Skeletal Muscle Tissue Engineering. RSC Adv. 2013, 3, 17938–17944. 10.1039/c3ra43499k.

[ref18] Ribeiro C.; Moreira S.; Correia V.; Sencadas V.; Rocha J. G.; Gama F. M.; Gomez-Ribelles J. L.; Lanceros-Mendez S. Enhanced Proliferation of Pre-osteoblastic Cells by Dynamic Piezoelectric Stimulation. RSC Adv. 2012, 2, 11504–11509. 10.1039/c2ra21841k.

[ref19] Ribeiro S.; Ribeiro C.; Carvalho E. O.; Tubio C. R.; Castro N.; Pereira N.; Correia V.; Gomes A. C.; Lanceros-Méndez S. Magnetically Activated Electroactive Microenvironments for Skeletal Muscle Tissue Regeneration. ACS Appl. Bio Mater. 2020, 3, 4239–4252. 10.1021/acsabm.0c00315. 35025425

[ref20] Guillot-Ferriols M.; Rodríguez-Hernández J. C.; Correia D. M.; Carabineiro S. A. C.; Lanceros-Méndez S.; Gómez Ribelles J. L.; Gallego Ferrer G. Poly(vinylidene) fluoride membranes coated by heparin/collagen layer-by-layer, smart biomimetic approaches for mesenchymal stem cell culture. Mater. Sci. Eng., C 2020, 117, 111281 10.1016/j.msec.2020.111281. 32919642

[ref21] Martins P. M.; Ribeiro S.; Ribeiro C.; Sencadas V.; Gomes A. C.; Gama F. M.; Lanceros-Méndez S. Effect of poling state and morphology of piezoelectric poly(vinylidene fluoride) membranes for skeletal muscle tissue engineering. RSC Adv. 2013, 3, 17938–17944. 10.1039/c3ra43499k.

[ref22] Maciel M. M.; Ribeiro S.; Ribeiro C.; Francesko A.; Maceiras A.; Vilas J. L.; Lanceros-Méndez S. Relation between fiber orientation and mechanical properties of nano-engineered poly(vinylidene fluoride) electrospun composite fiber mats. Composites, Part B 2018, 139, 146–154. 10.1016/j.compositesb.2017.11.065.

[ref23] Kitsara M.; Blanquer A.; Murillo G.; Humblot V.; De Bragança Vieira S. B.; Nogués C.; Ibáñez E.; Esteve J.; Barrios L. Permanently Hydrophilic, Piezoelectric PVDF Nanofibrous Scaffolds Promoting Unaided Electromechanical Stimulation on Osteoblasts. Nanoscale 2019, 11, 8906–8917. 10.1039/c8nr10384d. 31016299

[ref24] Esmaeili E.; Soleimani M.; Ghiass M. A.; Hatamie S.; Vakilian S.; Zomorrod M. S.; Sadeghzadeh N.; Vossoughi M.; Hosseinzadeh S. Magnetoelectric nanocomposite scaffold for high yield differentiation of mesenchymal stem cells to neural-like cells. J. Cell. Physiol. 2019, 234, 13617–13628. 10.1002/jcp.28040. 30613971 PMC13483200

[ref25] Wu S.; Chen M.-S.; Maurel P.; Lee Y.; Bunge M. B.; Arinzeh T. L. Aligned Fibrous PVDF-TrFE Scaffolds with Schwann Cells Support Neurite Extension and Myelination in Vitro. J. Neural. Eng. 2018, 15, 056010 10.1088/1741-2552/aac77f. 29794323 PMC6125183

[ref26] Hitscherich P.; Wu S.; Gordan R.; Xie L.; Arinzeh T.; Lee E. J. The effect of PVDF-TrFE scaffolds on stem cell derived cardiovascular cells. Biotechnol. Bioeng. 2016, 113, 1577–1585. 10.1002/bit.25918. 26705272

[ref27] Correia D. M.; Gonçalves R.; Ribeiro C.; Sencadas V.; Botelho G.; Ribelles J. L.; Lanceros-Méndez S. Electrosprayed poly(vinylidene fluoride) microparticles for tissue engineering applications. RSC Adv. 2014, 4, 33013–33021. 10.1039/c4ra04581e.

[ref28] Sobreiro-Almeida R.; Tamaño-Machiavello M. N.; Carvalho E. O.; Cordón L.; Doria S.; Senent L.; Correia D. M.; Ribeiro C.; Lanceros-Méndez S.; Sabater i Serra R.; Gomez Ribelles J. L.; Sempere A. Human Mesenchymal Stem Cells Growth and Osteogenic Differentiation on Piezoelectric Poly(vinylidene fluoride) Microsphere Substrates. Int. J. Mol. Sci. 2017, 18, 2391 10.3390/ijms18112391. 29137121 PMC5713360

[ref29] Hermenegildo B.; Ribeiro C.; Pérez-Álvarez L.; Vilas J. L.; Learmonth D. A.; Sousa R. A.; Martins P.; Lanceros-Méndez S. Hydrogel-based Magnetoelectric Microenvironments for Tissue Stimulation. Colloids Surf., B 2019, 181, 1041–1047. 10.1016/j.colsurfb.2019.06.023. 31382332

[ref30] Fernandes M. M.; Correia D. M.; Ribeiro C.; Castro N.; Correia V.; Lanceros-Mendez S. Bioinspired Three-dimensional Magnetoactive Scaffolds for Bone Tissue Engineering. ACS Appl. Mater. Interfaces 2019, 11, 45265–45275. 10.1021/acsami.9b14001. 31682095

[ref31] Damaraju S. M.; Shen Y.; Elele E.; Khusid B.; Eshghinejad A.; Li J.; Jaffe M.; Arinzeh T. L. Three-dimensional Piezoelectric Fibrous Scaffolds Selectively Promote Mesenchymal Stem Cell Differentiation. Biomaterials 2017, 149, 51–62. 10.1016/j.biomaterials.2017.09.024. 28992510

[ref32] Kitsara M.; Dufour T.; Humblot V.; Legris M.; Simon A.; Revert G.; Agbulot O. Plasma-modification of PVDF Scaffolds Improve Cardiomyocytes Viability and Morphology. Book of Abstracts, 2019 Annual Meeting & Exposition of Society for Biomaterials: Seattle, WA, 2017.

[ref33] Correia D. M.; Goncalves R.; Ribeiro C.; Sencadas V.; Botelho G.; Gomez-Ribelles J. L.; Lanceros-Mendez S. Electrosprayed Poly (Vinylidene fluoride) Microparticles for Tissue Engineering Applications. RSC Adv. 2014, 4, 33013–33021. 10.1039/c4ra04581e.

[ref34] Costa L. M. M.; Bretas R. E. S.; Gregorio R. Effect of Solution Concentration on the Electrospray/Electrospinning Transition and on the Crystalline Phase of PVDF. Mater. Sci. Appl. 2010, 01, 247 10.4236/msa.2010.14036.

[ref35] Macedo A. S.; Carvalho E. O.; Cardoso V. F.; Correia D. M.; Tubio C. R.; Fidalgo-Marijuan A.; Botelho G.; Lanceros-Méndez S. Tailoring electroactive poly(vinylidene fluoride-co-trifluoroethylene) microspheres by a nanoprecipitation method. Mater. Lett. 2020, 261, 127018 10.1016/j.matlet.2019.127018.

[ref36] Dong M.; Hafezi M.; Tong Z.; Qin L. Preparation and Oil Lubrication of Polyvinylidene fluoride (PVDF) Nanospheres. Mater. Res. Express 2019, 6, 085093 10.1088/2053-1591/ab220e.

[ref37] Whitesides G. M. The Origins and the Future of Microfluidics. Nature 2006, 442, 368 10.1038/nature05058. 16871203

[ref38] Teh S.-Y.; Lin R.; Hung L.-H.; Lee A. P. Droplet Microfluidics. Lab Chip 2008, 8, 198–220. 10.1039/b715524g. 18231657

[ref39] Xu S.; Nie Z.; Seo M.; Lewis P.; Kumacheva E.; Stone H. A.; Garstecki P.; Weibel D. P.; Gitlin I.; Whitesides G. M. Generation of Monodisperse Particles by Using Microfluidics: Control Over Size, Shape, and Composition. Angew. Chem., Int. Ed. 2005, 44, 724–728. 10.1002/anie.200462226. 15612064

[ref40] Serra C. A.; Chang Z. Microfluidic-Assisted Synthesis of Polymer Particles. Chem. Eng. Technol. 2008, 31, 1099–1115. 10.1002/ceat.200800219.

[ref41] Brzeziński M.; Socka M.; Kost B. Microfluidics for Producing Polylactide Nanoparticles and Microparticles and their Drug Delivery Application. Polym. Int. 2019, 68, 997–1014. 10.1002/pi.5753.

[ref42] Choi C.-H.; Jung J.-H.; Rhee Y. W.; Kim D.-P.; Shim S.-E.; Lee C.-S. Generation of Monodisperse Alginate microbeads and in Situ Encapsulation of Cell in Microfluidic Device. Biomed. Microdevices 2007, 9, 855–862. 10.1007/s10544-007-9098-7. 17578667

[ref43] Heshmatnezhad F.; Solaimany Nazar A. R. S. On-chip Controlled Synthesis of Polycaprolactone Nanoparticles using Continuous-flow Microfluidic Devices. J. Flow Chem. 2020, 10, 533–543. 10.1007/s41981-020-00092-8.

[ref44] Xiong L.; Chen P.; Zhou Q. Adhesion Promotion Between PDMS and Glass by Oxygen Plasma Pre-treatment. J. Adhes. Sci. Technol. 2014, 28, 1046–1054. 10.1080/01694243.2014.883774.

[ref45] Martins P.; Costa C. M.; Costa M.; Benelmekki G.; Botelho S.; Lanceros-Mendez S. On the origin of the electroactive poly(vinylidene fluoride) β-phase nucleation by ferrite nanoparticles via surface electrostatic interactions. CrystEngComm 2012, 14, 2807–2811. 10.1039/c2ce06654h.

[ref46] Vaca-González J. J.; Clara-Trujillo S.; Guillot-Ferriols M.; Ródenas-Rochina J.; Sanchis M. J.; Ribelles J. L.; Garzón-Alvarado D. A.; Ferrer G. Effect of electrical stimulation on chondrogenic differentiation of mesenchymal stem cells cultured in hyaluronic acid - Gelatin injectable hydrogels. Bioelectrochemistry 2020, 134, 107536 10.1016/j.bioelechem.2020.107536. 32335352

[ref47] Seemann R.; Brinkmann M.; Pfohl T.; Herminghaus S. Droplet Based Microfluidics. Rep. Prog. Phys. 2011, 75, 016601 10.1088/0034-4885/75/1/016601. 22790308

[ref48] Gao Y.; Zhao C.-X.; Sainsbury F. Droplet Shape Control using Microfluidics and Designer Biosurfactants. J. Colloid Interface Sci. 2021, 584, 528–538. 10.1016/j.jcis.2020.09.126. 33129162

[ref49] Cardoso V. F.; Botelho G.; Lanceros-Méndez S. Nonsolvent induced phase separation preparation of poly(vinylidene fluoride-co-chlorotrifluoroethylene) membranes with tailored morphology, piezoelectric phase content and mechanical properties. Mater. Des. 2015, 88, 390–397. 10.1016/j.matdes.2015.09.018.

[ref50] Ribeiro C.; Costa C. M.; Correia D. M.; Nunes-Pereira J.; Oliveira J.; Martins P.; Gonçalves R.; Cardoso V. F.; Lanceros-Méndez S. Electroactive poly(vinylidene fluoride)-based structures for advanced applications. Nat. Protoc. 2018, 13, 681 10.1038/nprot.2017.157. 29543796

[ref51] Cai X.; Lei T.; Sun D.; Lin L. A critical analysis of the α, β and γ phases in poly(vinylidene fluoride) using FTIR. RSC Adv. 2017, 7, 15382–15389. 10.1039/c7ra01267e.

[ref52] Martins P.; Lopes A. C.; Lanceros-Mendez S. Electroactive phases of poly(vinylidene fluoride): Determination, processing and applications. Prog. Polym. Sci. 2014, 39, 683–706. 10.1016/j.progpolymsci.2013.07.006.

[ref53] Ruan L.; Yao X.; Chang Y.; Zhou L.; Qin G.; Zhang X. Properties and Applications of the β Phase Poly(vinylidene fluoride). Polymers 2018, 10, 228 10.3390/polym10030228. 30966263 PMC6415445

[ref54] Lizundia E.; Reizabal A.; Costa C. M.; Maceiras A.; Lanceros-Méndez S. Electroactive γ-Phase, Enhanced Thermal and Mechanical Properties and High Ionic Conductivity Response of Poly (Vinylidene Fluoride)/Cellulose Nanocrystal Hybrid Nanocomposites. Materials 2020, 13, 743 10.3390/ma13030743. 32041217 PMC7040804

[ref55] Barrau S.; Ferri A.; Da Costa A.; Defebvin J.; Leroy S.; Desfeux R.; Lefebvre J. M. Nanoscale Investigations of α- and γ-Crystal Phases in PVDF-Based Nanocomposites. ACS Appl. Mater. Interfaces 2018, 10, 13092–13099. 10.1021/acsami.8b02172. 29589902

[ref56] Prest W. M.; Luca D. J. The Formation of the γ-Phase from the α and β Polymorphs of Polyvinylidene Fluoride. J. Appl. Phys. 1978, 49, 5042–5047. 10.1063/1.324439.

[ref57] Hasegawa R.; Kobayashi M.; Tadokoro H. Molecular Conformation and Packing of Poly(vinylidene fluoride). Stability of Three Crystalline Forms and the Effect of High Pressure. Polym. J. 1972, 3, 591–599. 10.1295/polymj.3.591.

[ref58] Nishiyama T.; Sumihara T.; Sasaki Y.; Sato E.; Yamato M.; Horibe H. Crystalline structure control of poly(vinylidene fluoride) films with the antisolvent addition method. Polym. J. 2016, 48, 1035–1038. 10.1038/pj.2016.62.

[ref59] Kim K. M.; Jeon W. S.; Park N.-G.; Ryu K. S.; Chang S. H. Effect of evaporation temperature on the crystalline properties of solution-cast films of poly(vinylidene fluoride)s. Korean J. Chem. Eng. 2003, 20, 934–941. 10.1007/bf02697302.

[ref60] Martins P.; Lasheras A.; Gutierrez J.; Barandiaran J. M.; Orue I.; Lanceros-Mendez S. Optimizing piezoelectric and magnetoelectric responses on CoFe2O4/P(VDF-TrFE) nanocomposites. J. Phys. D: Appl. Phys. 2011, 44, 495303 10.1088/0022-3727/44/49/495303.

[ref61] Lopes A. C.; Costa C. M.; Tavares C. J.; Neves I. C.; Lanceros-Mendez S. Nucleation of the Electroactive γ Phase and Enhancement of the Optical Transparency in Low Filler Content Poly(vinylidene)/Clay Nanocomposites. J. Phys. Chem. C 2011, 115, 18076–18082. 10.1021/jp204513w.

[ref62] Bliznyuk V. N.; Baig A.; Singamaneni S.; Pud A. A.; Fatyeyeva K. Y.; Shapoval G. S. Effects of surface and volume modification of poly(vinylidene fluoride) by polyaniline on the structure and electrical properties of their composites. Polymer 2005, 46, 11728–11736. 10.1016/j.polymer.2005.09.058.

[ref63] Wojnarowska Z.; Grzybowska K.; Hawelek L.; Dulski M.; Wrzalik R.; Gruszka I.; Paluch M.; Pienkowska K.; Sawicki W.; Bujak P.; Paluch K. J.; Tajber L.; Markowski J. Molecular Dynamics, Physical Stability and Solubility Advantage from Amorphous Indapamide Drug. Mol. Pharm. 2013, 10, 3612–3627. 10.1021/mp400116q. 24070615

[ref64] Correia D. M.; Costa C. M.; Rodríguez Hernández J. C.; Tort-Ausina I.; Biosca L. T.; Torregrosa Cabanilles C. T.; Meseguer-Dueñas J.; Krakovsky I.; Lanceros-Méndez S.; Gomez-Ribelles J. L.; Gómez Ribelles J. L. Crystallization Monitoring of Semicrystalline Poly(vinylidene fluoride)/1-Ethyl-3-methylimidazolium Hexafluorophosphate [Emim][PF6] Ionic Liquid Blends. Cryst. Growth Des. 2021, 21, 4406–4416. 10.1021/acs.cgd.1c00333.

[ref65] Correia D. M.; Costa C. M.; Lizundia E.; Sabater i Serra R.; Gómez-Tejedor J. A.; Biosca L. T.; Meseguer-Dueñas J.; Gomez Ribelles J. L.; Lanceros-Méndez S. Influence of Cation and Anion Type on the Formation of the Electroactive β-Phase and Thermal and Dynamic Mechanical Properties of Poly(vinylidene fluoride)/Ionic Liquids Blends. J. Phys. Chem. C 2019, 123, 27917–27926. 10.1021/acs.jpcc.9b07986.

[ref66] Salmerón Sánchez M.; Mathot V. B. F.; Vanden Poel G.; Gómez Ribelles J. L. Effect of the Cooling Rate on the Nucleation Kinetics of Poly (L-Lactic acid) and its Influence on Morphology. Macromolecules 2007, 40, 7989–7997. 10.1021/ma0712706.

[ref67] Gregorio R. Jr Determination of the α, β, and γ crystalline phases of poly(vinylidene fluoride) films prepared at different conditions. J. Appl. Polym. Sci. 2006, 100, 3272–3279. 10.1002/app.23137.

[ref68] Ourry L.; Marchesini S.; Bibani M.; Mercone S.; Ammar S.; Mammeri F. Influence of nanoparticle size and concentration on the electroactive phase content of PVDF in PVDF-CoFe2O4-based hybrid films. Phys. Status Solidi A 2015, 212, 252–258. 10.1002/pssa.201431563.

[ref69] Botelho G.; Lanceros-Mendez S.; Gonçalves A. M.; Sencadas V.; Rocha J. G. Relationship between processing conditions, defects and thermal degradation of poly(vinylidene fluoride) in the β-phase. J. Non-Cryst. Solids 2008, 354, 72–78. 10.1016/j.jnoncrysol.2007.07.012.

[ref70] Gonçalves R.; Martins P. M.; Caparros J.; Martins P.; Benelmekki M.; Botelho G.; Lanceros-Mendez S.; Lasheras A.; Gutierrez J.; Barandiaran J. M. Nucleation of the Electroactive β-Phase, Dielectric and Magnetic Response of Poly (Vinylidene fluoride) Composites with Fe2O3 Nanoparticles. J. Non-Cryst. Solids 2013, 361, 93–99. 10.1016/j.jnoncrysol.2012.11.003.

[ref71] Mitsuda K.; Kimura H.; Murahashi T. Evaporation and Decomposition of Triton X-100 under Various Gases and Temperatures. J. Mater. Sci. 1989, 24, 413–419. 10.1007/bf01107420.

[ref72] Cho K.-H.; Bichurin M. I.; Petrov V. M.; Bhalla A.; Priya S. Magnetoelectric Laminate Composite: Effect of Piezoelectric Layer on Magnetoelectric Properties. Ferroelectrics 2014, 473, 110–128. 10.1080/00150193.2014.923670.

[ref73] Wickens A.; Robinson J. Magnetoelectric Neural Modulation. Biophys. J. 2017, 112, 286 10.1016/j.bpj.2016.11.1549.

