# Supplementary material for: Microfluidic Processing of Piezoelectric and Magnetic Responsive Electroactive Microspheres
Source: ACS Appl Polym Mater. 2022 Jul 29;4(8):5368–79. doi: 10.1021/acsapm.2c00380 (PMC9940114; doi:10.1021/acsapm.2c00380)
Supplement: Supplementary file 1 — ap2c00380_si_001.pdf [file ap2c00380_si_001.pdf]

Supporting Information

Microfluidics processing of piezoelectric and magnetic responsive electroactive microspheres

Luís Amaro Martins[1], Joaquín Ródenas-Rochina[1], Daniel Salazar[2], Vanessa F. Cardoso[3,4], José Luis Gómez Ribelles[1,5*] & Senentxu Lanceros-Mendez[2,6*]

1 *CBIT – Centre for Biomaterials and Tissue Engineering, Universitat Politècnica de València, Valencia 46022, Spain; *jlgomez@ter.upv.es*
2 *BCMaterials, Basque Center for Materials Applications and Nanostructures, UPV/EHU Science Park, Leioa 48940, Spain; *senentxu.lanceros@bcmaterials.net (S.L.M.)*
3 *Department of Physics, Universidade do Minho, Braga 4710-057, Portugal*
4 *CMEMS-UMinho, Universidade do Minho, Guimarães 4800-058, Portugal*
5 *Biomedical Research Networking Center on Bioengineering, Biomaterials, and Nanomedicine (CIBER-BBN), Madrid 28029, Spain*
6 *IKERBASQUE, Basque Foundation for Science, Bilbao 48009, Spain*

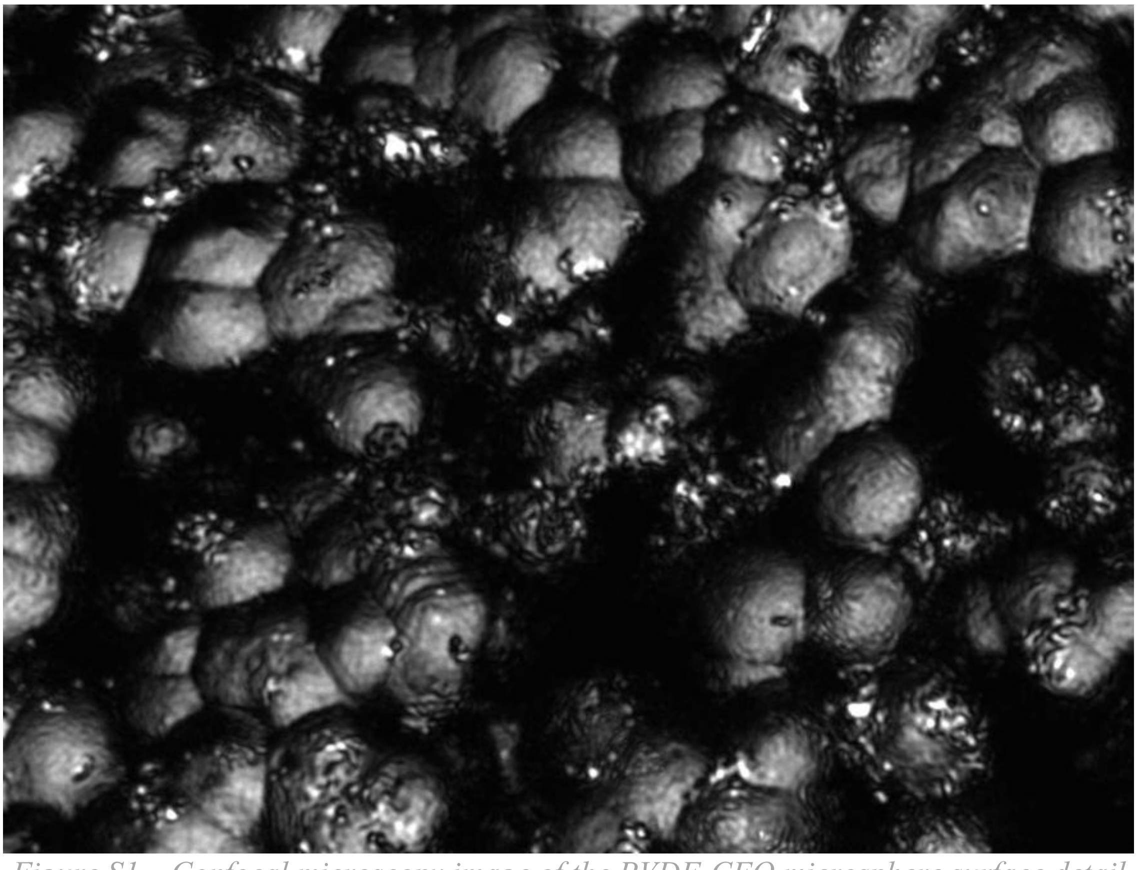

Figure S1 – Confocal microscopy image of the PVDF-CFO microsphere surface detail

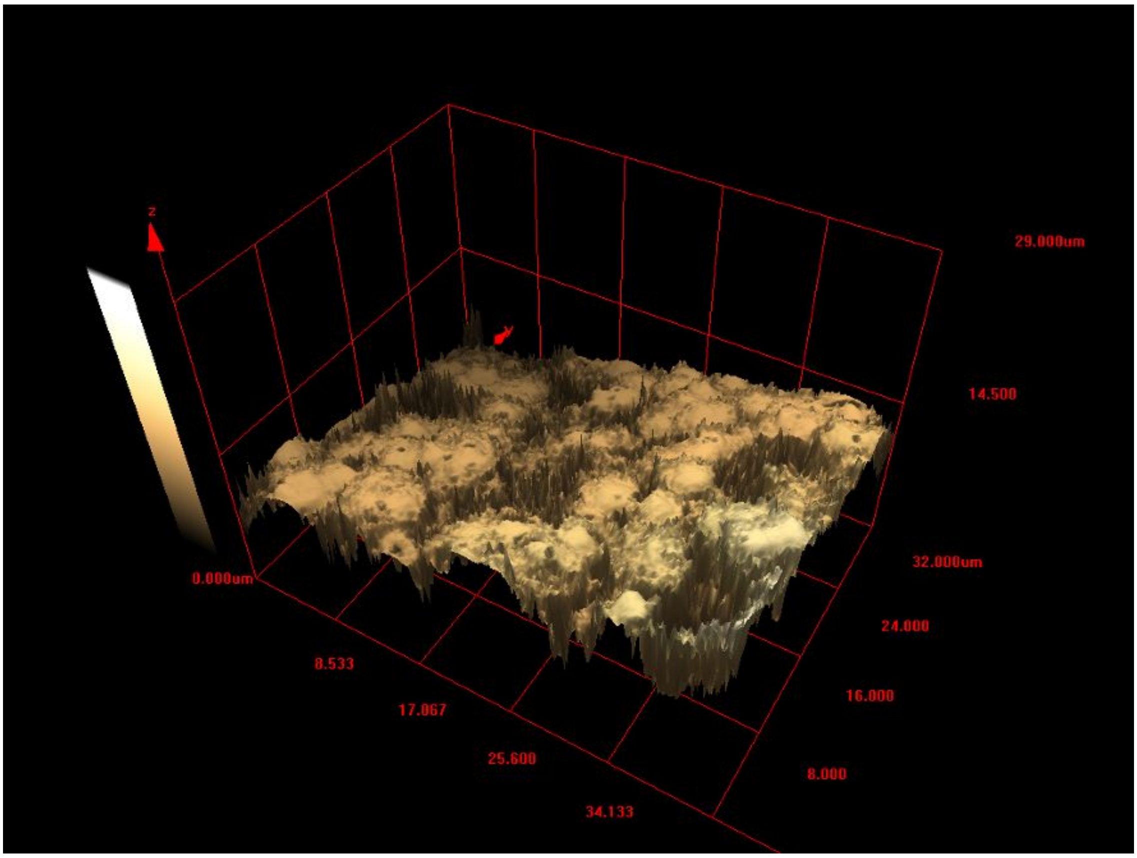

Figure S2 – Confocal microscopy reconstruction of the PVDF-CFO microspheres surface structure

Acknowledgments

This work has been funded by the Spanish State Research Agency (AEI) through the projects PID2019-106099RB-C41 and C43/AEI/10.13039/501100011033. CIBER-BBN is an initiative funded by the VI National R&D&I Plan 2008–2011, Iniciativa Ingenio 2010, Consolider Program. CIBER Actions are financed by the Instituto de Salud Carlos III with assistance from the European Regional Development Fund. V.F.C. also thanks to the Portuguese Foundation for Science and Technology (FCT) for financial support under the Assistant Researcher Contract 2020.02304.CEECIND. The authors also acknowledge funding from the Basque Government Industry and Education Departments under the ELKARTEK and PIBA (PIBA-2018-06) programs, respectively.

The author also thanks to the microscopy service of the Universitat Politècnica de València and Jose Garcia Anton and Dionisio Miguel Garcia Garcia from the Chemical Engineering Department for all the assistance, patience and attention in acquiring microscopy imagens.
